# Pharmacist led homeless outreach engagement and non-medical independent prescribing (Rx) (PHOENIx) intervention for people experiencing homelessness: a non- randomised feasibility study

**DOI:** 10.1186/s12939-020-01337-7

**Published:** 2021-01-07

**Authors:** Richard Lowrie, Kate Stock, Sharon Lucey, Megan Knapp, Andrea Williamson, Margaret Montgomery, Cian Lombard, Donogh Maguire, Rachael Allan, Rebecca Blair, Vibhu Paudyal, Frances S. Mair

**Affiliations:** 1 Homeless Health, Pharmacy Services, Clarkston Court, NHS Greater Glasgow & Clyde, 56 Busby Road, Clarkston, Glasgow, G76 7AT UK; 2 Simon Community Scotland, Glasgow, UK; 3 Department of General Practice and Primary Care, Institute of Health and Wellbeing, University of Glasgow, Glasgow, UK; 4 Acute Homeless Liaison Team, NHS Greater Glasgow and Clyde, Glasgow, UK; 5 Emergency Department, Glasgow Royal Infirmary, NHS Greater Glasgow & Clyde, Glasgow, UK; 6 Homelessness Health Service, Hunter St, Glasgow, UK; 7 University of Birmingham, Birmingham, UK

**Keywords:** Homelessness inequality policy service, Pharmacist, Prescribing

## Abstract

**Background:**

Homelessness and associated mortality and multimorbidity rates are increasing. Systematic reviews have demonstrated a lack of complex interventions that decrease unscheduled emergency health services utilisation or increase scheduled care. Better evidence is needed to inform policy responses. We examined the feasibility of a complex intervention (PHOENIx: Pharmacist led Homeless Outreach Engagement Nonmedical Independent prescribing (Rx)) to inform a subsequent pilot randomised controlled trial (RCT).

**Methods:**

Non-randomised trial with Usual Care (UC) comparator group set in Greater Glasgow and Clyde Health Board, Scotland**.** Participants were adult inpatients experiencing homelessness in a city centre Glasgow hospital, referred to the PHOENIx team at the point of hospital discharge, from 19th March 2018 until 6th April 2019. The follow up period for each patient started on the day the patient was first seen (Intervention group) or first referred (UC), until 24th August 2019, the censor date for all patients. All patients were offered and agreed to receive serial consultations with the PHOENIx team (NHS Pharmacist prescriber working with Simon Community Scotland (third sector homeless charity worker)). Patients who could not be reached by the PHOENIx team were allocated to the UC group. The PHOENIx intervention included assessment of physical/mental health, addictions, housing, benefits and social activities followed by pharmacist prescribing with referral to other health service specialities as necessary. All participants received primary (including specialist homelessness health service based general practitioner care, mental health and addictions services) and secondary care. Main outcome measures were rates of: recruitment; retention; uptake of the intervention; and completeness of collected data, from recruitment to censor date.

**Results:**

Twenty four patients were offered and agreed to participate; 12 were reached and received the intervention as planned with a median 7.5 consultations (IQR3.0–14.2) per patient. The pharmacist prescribed a median of 2 new (IQR0.3–3.8) and 2 repeat (1.3–7.0) prescriptions per patient; 10(83%) received support for benefits, housing or advocacy. Twelve patients were not subsequently contactable after leaving hospital, despite agreeing to participate, and were assigned to UC. Two patients in the UC group died of drug/alcohol overdose during follow up; no patients in the Intervention group died. All 24 patients were retained in the intervention or UC group until death or censor date and all patient records were accessible at follow up: 11(92%) visited ED in both groups, with 11(92%) hospitalisations in intervention group, 9(75%) UC. Eight (67%) intervention group patients and 3(25%) UC patients attended scheduled out patient appointments.

**Conclusions:**

Feasibility testing of the PHOENIx intervention suggests merit in a subsequent pilot RCT.

## Introduction

Homelessness encompasses rough sleeping, living in unsuitable or temporary accommodation e.g. shelters, or sofa surfing [1]. The risks of experiencing homelessness are based on a set of individual and structural factors such as childhood poverty, experiencing abuse, a lack of support or lack of affordable housing, most of which are out with the control of the individual [2]. The prevalence of homelessness has increased since 2016 when there were 34,100 homeless applications: in 2018–19 homeless applications increased to 36,465 (0.7% of the Scottish population). Glasgow experienced the greatest increase in Scotland, from 5274 in 2018 to 5873 (0.93% of the Glasgow population) in 2019 [3]. In England, rates of rough sleeping have doubled in the past 6 years, [4] impacting on health and mortality [5–7]. In Scotland, all health service provision is free at the point of access and there are no financial barriers to care; prescribed medicines are also free. Access to primary care services remains challenging due to stigma, discrimination and bureaucratic barriers [8, 9]. Unmet health needs are common [10, 11]. Mental illness, pain and addictions may be poorly managed [12] with evidence suggesting under prescribing and low rates of prescription adherence [13, 14] Patterns of emergency healthcare utilisation differ between people experiencing homelessness and those who are housed: ED visits, hospitalisations and readmissions are higher [15, 16]. Reasons also differ, with people who are homeless experiencing more emergency healthcare contacts for drug, alcohol and mental health related problems [15, 16].

Availability and uptake of preventative healthcare is lower [9, 16, 17] and presentations to Family (General) Practitioners tend to be reactive [18]. Almost one third of homeless deaths are preventable by timely and effective use of health services [6] yet there is a lack of community based effective and cost effective complex interventions by healthcare professionals, proven to increase anticipatory care and reducing unscheduled care [19]. In Glasgow, Scotland, a novel, low threshold Pharmacist led Homeless Outreach Engagement and Nonmedical Independent prescribing (Rx) (PHOENIx) service is offered to people experiencing homelessness, by NHS (National Health Service) employee Pharmacist independent prescribers working with Simon Community Scotland (SCS) Outreach workers (third sector homelessness charity expert in housing assessment, benefits, advocacy and social prescribing) in city centre venues [20–23]. Aiming to assess and address housing, health, benefits and social activities, the views of people experiencing homelessness, and stakeholders (e.g. General Practitioners, Addictions workers, community pharmacists, Hostel and Day Centre workers), of the PHOENIx intervention are favourable [24]. In line with the development of complex interventions, [25] the aim of this study was to examine the feasibility of recruitment, retention, the extent of intervention delivery, and collection of outcome data [26] prior to embarking on a pilot randomised controlled trial.

## Methods

### Design

Feasibility and pilot studies are essential precursors to full scale trials of complex health service / public health interventions. Feasibility studies precede pilot studies. Feasibility studies look at specific aspects of the design, and pilot studies aim to test whether the entire procedures of the full study work together [25, 26].

### Patient inclusion

Participants were adult inpatients (≥ 18 years) in a Glasgow city centre hospital, at the point of discharge into the community, who were experiencing homelessness, and either had no registered GP or were registered with Glasgow’s Homelessness Health Service. In general, Scottish hospitals, unlike those in England, [27] have no specialist ‘step down’ intermediate care service to help reduce readmissions.

We aimed to recruit the same number of intervention and usual care group patients. As part of routine service provision, the hospital based Acute Homeless Liaison Team (AHLT) obtained patient consent for referral and follow up by the PHOENIx team, then referred patients (by NHS email), to the PHOENIx team. AHLT engage with patients in hospital to try to construct a plan for their discharge back into the community. AHLT supplied mobile phones or top up vouchers to patients to facilitate contact. On receiving a referral from AHLT, but before contacting the patient, the NHS Pharmacist accessed the patient’s shared clinical and prescribing records to collect relevant baseline information. The PHOENIx team then phoned the patient (if a number was available) or visited or phoned their place of residence or visited their begging pitch or one of Glasgow city centre’s low threshold homeless day shelters, to arrange and conduct the first face to face consultation.

### PHOENIx team

All four PHOENIx staff, in addition to their expert professional skills and experience, were recruited based on their contextual knowledge, street sense, an attitude of mutual respect and empathy for patients, and resilience. The team had an ideology of care including social inclusion, and unconditional support [28]. Consultations with patients involved a Pharmacist and a SCS worker. Pharmacists were NHS employees, and SCS employed outreach workers held honorary NHS contracts. The pharmacists had at least 2 years’ experience as Independent Prescribers, and were familiar with assessing and managing the complex needs of people experiencing homelessness. Pharmacist independent prescribers have undergone additional training in therapeutics and learning in practice, then passed an exam and have additional registration with the General Pharmaceutical Society to legally prescribe any medicine within their sphere of competency. An independent prescriber can assess, diagnose and prescribe without the need to involve another healthcare professional. The scope of practice depends on the training received and the eventual role. Pharmacists working in a hospital intensive care unit may be expected to independently prescribe any parenteral medicines; whereas a pharmacist working in an out-patient rheumatology clinic would be expected to independently prescribe a range of anti-rheumatic medicines. Independent prescribing pharmacists working in homeless health are expected to be adept at prescribing a wide range of medicines, given the number of morbidities experienced by people who are homeless [13, 18]. They access the additional input of the general practitioners in homelessness health or other specialists when required [13, 18].

SCS outreach staff had at least 2 years’ experience caring for people experiencing homelessness, part of a larger SCS outreach team in Glasgow City Centre. They were trained in housing and benefits assessment and had a detailed knowledge of rough sleeping sites and night shelters in Glasgow city centre. One day per week of pharmacist time was allocated to the service and 3 days of SCS outreach worker time.

### Intervention

**Fig. 1:**
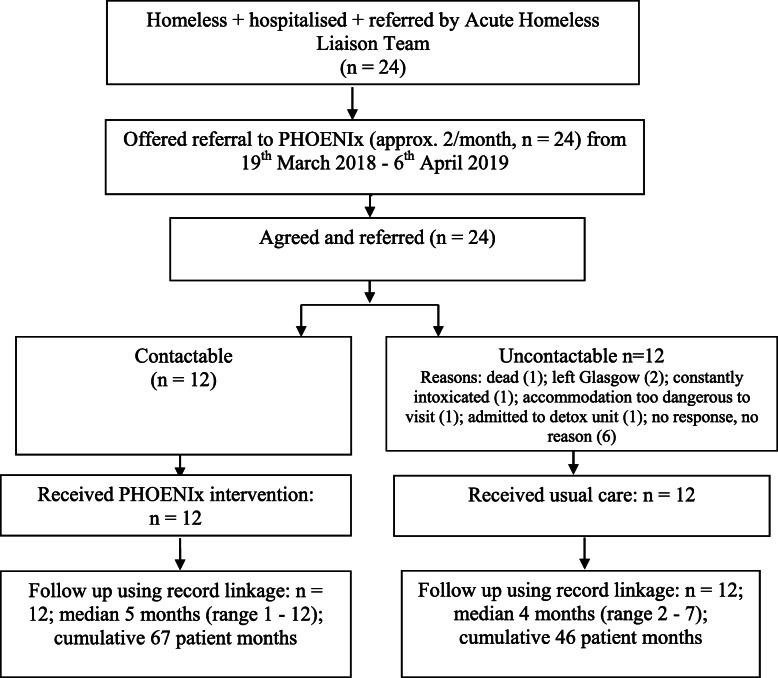
Flow of patients during the study

As described previously, and as shown in Fig. 1, the pharmacist and SCS worker consulted with the patient to work through a comprehensive health check during which they assessed, and where possible, began or continued treatment for: physical or mental health problems and addictions (Table 1) [20, 22]. Housing, benefits and social care needs were also assessed. In the case of treatment for opioid dependency, patients were referred to the relevant specialist service e.g. for same day initiation of methadone. Prescribing of all other medicines was undertaken by the pharmacist, after checking relevant clinical records for evidence of previous recent supply, or after establishing a new diagnoses needing treatment e.g. wound infection. The PHOENIx team and the patient agreed the frequency of return consultations. A low caseload (less than 10 patient visits per working day) enabled sufficient time to offer repeated outreach visits, and minimised the risk of staff being overwhelmed in the process of supporting patients with multiple complex needs including, in most cases, traumatic experiences that the patient may or may not have shared previously. The team accepted the patient’s priorities and progressed at the patient’s pace, for example, if the patient had untreated hepatitis C infection but wanted to prioritise dental treatment, the team worked with the patient and dental support worker, to engage the patient in dental treatment. The pharmacist remotely accessed and updated relevant NHS clinical patient data systems during each consultation, and the SCS worker remotely accessed and updated relevant social care and third sector care records.

**Table 1:** PHOENIx intervention and usual care^a^

Timeline	Activity	
19th March 2018 - 24th August 2019	**Intervention:** patient access to GP, hospital and other services as normal. Plus PHOENIx consultations assessing: ˗ Quality of Life; ˗ Accommodation; ˗ Debt; ˗ Social activity/interests; ˗ Cardiovascular disease; ˗ Respiratory disease ˗ Nutrition screen; ˗ Footcare; ˗ Drug and alcohol use; ˗ Prescribed medicines and review; ˗ Mental health; ˗ Blood Borne Viruses; ˗ Sexual health; ˗ Wounds; ˗ Fractures; ˗ Teeth; ˗ Eyesight; ˗ Skin. Following assessment, the patient prioritises their own issues, and the PHOENIx team treat, prescribe and refer where appropriate. A plan for the next visit is agreed.	**Usual care:** patient access to GP, hospital and other services as normal.

^a^at least four unsuccessful phone calls over four days, and cannot be traced in Glasgow City centre streets/homeless accommodation

### Usual care

Patients who could not be reached despite at least four phone calls over 4 days and who could not be traced in Glasgow City centre streets/homeless accommodation were deemed uncontactable and assigned to the UC group. Patients in both groups continued to receive health and social care and medicines as usual from all existing providers.

UC for patients who are homeless and discharged from hospital comprised a referral letter sent electronically to the patient’s registered GP, summarising the admission diagnoses and treatments given, with a note of the patient’s 7 day discharge prescription (if any) and planned follow up (if any). The patient received a copy of this, and was expected to attend or contact their GP practice for a continuation prescription of these medicines if indicated, and for follow up care. If the patient had an addiction, the addiction team in the hospital communicated with the addiction team in primary care, to ensure continuation of supply of treatments for addictions e.g. methadone or buprenorphine for opiate addiction.

Glasgow’s Homelessness Health Service registers people who are homeless, if the patient attends the practice. It provides comprehensive primary care (GP and Nursing services, plus referral to other services who visit the Homelessness Health Service at fixed times every week e.g. podiatry, dieticians, oral health, Blood Borne Virus Nurse specialists). The homelessness addiction and mental health services are co-located. People who are homeless are free to retain their registration with a mainstream GP practice rather than register with the Homelessness Health Service.

Glasgow has approximately 38 discrete temporary locations for multiple occupancy temporary accommodation for people who are homeless, providing approximately 800 beds. For those people in Glasgow who are homeless and accommodated by Glasgow City Council services, accommodation ranges from those with 24 h staff supervision (for patients detoxing from alcohol/drugs or both) to Bed and Breakfasts with no supervision or cooking facilities.

### Outcomes

Outcome measures were: rates of recruitment; retention, uptake of the intervention (proportion of patients receiving the intervention as planned) and the extent of collection of baseline and outcome data (number and type of Emergency Department (ED) visits and hospitalisations differentiated by physical, mental ill health or addictions; proportion of out-patient appointment attendances). The period of observation for outcome data was the period from 19th March 2018 (the date of referral into the service (UC group patients) or date of first contact (intervention group patients) until the censor date of 24th Aug 2019 or death if sooner.

### Data

Data were assessed using electronic surveillance of medical records for all participants. An NHS employee researcher with read only access to clinical systems transcribed relevant data onto an EXCEL spreadsheet, stored on a password protected NHS desktop computer, ready for summary and analysis. All transcribed data were checked for accuracy and completeness. Pre-specified baseline data included demographics, accommodation type, registration with GP/other services and acute services attendance in the past year. Follow up data included changes implemented during consultations, and clinical outcomes (number and type of acute care contacts). Baseline data were obtained by looking up each patient’s clinical records, using their unique personal health number (Community Health Index (CHI) number). Actions resulting from intervention delivery were recorded by pharmacists and SCS workers as part of routine service delivery. Outcome data were collected as follows, from clinical records: when patients are discharged from ED or hospital, a summary letter including reason for attendance appears on the patient’s shared primary and secondary care clinical record (Clinical Portal). The primary reason for ED attendance was taken from the patient’s emergency attendance letter (‘presenting complaint’ or ‘diagnosis’ or ‘significant operations or procedures’) and reasons for hospitalisation taken from the immediate discharge letter (‘primary diagnosis’ or ‘secondary diagnosis’). These were categorised as either: physical health; mental health; or addictions by the NHS employee researcher. Where there was no reason given, the researcher accessed a separate, shared clinical record (Trakcare) for the same episode of care, to collect the ICD 10 code assigned to each episode of care (‘current diagnosis’ or ‘presenting complaint’). In cases where there was no coded diagnosis or ‘no injury or abnormality detected’ or no entry in the relevant TRAK section, the ‘clinical comments’ section of the immediate discharge letter or ‘reason for attendance’ was used.

### Statistical analyses

Continuous data were summarised using mean (SD) or median (IQR). For categorical data, percentages were calculated. All analyses were conducted by an NHS researcher and repeated by another member of the research team, using MINITAB version 18 [29].

## Results

### Patient characteristics at baseline

Twenty four patients (approximately 5 % of the eligible population during the study period) were offered and agreed to referral to the PHOENIx service, between 19th March 2018 and 6th April 2019. After referral, 12 patients could not be reached and therefore formed a UC group. Table 2 shows baseline demographics.

**Table 2:** Patient characteristics at baseline^a^

	Intervention ***n*** = 12	Usual care ***n*** = 12
**Demographics**		
Age (years)	42 (36–47)	39 (33.5–47.5)
Sex (male)	10 (83)	10 (83)
Ethnicity (white Scottish)	12 (100)	12 (100)
**Accommodation**		
Temporary e.g. hostel	9 (75)	5 (46)
Street	3 (25)	2 (17)
Sofa surfing	0 (0)	2 (17)
Unknown	0 (0)	3 (25)
**General Practitioner**		
Homeless Health Service	10 (83)	8 (67)
No recent GP	2 (17)	4 (33)
**Registration with other services**		
Homeless Addiction Team	11 (92)	6 (50)
City Ambition Network	3 (25)	2 (17)

^a^N (%) or median (IQR).

Nine (75%) patients in the intervention group and five (46%) in the UC group were discharged into temporary homeless accommodation; three in the intervention group and two in the UC group were rough sleeping after discharge. Eleven (92%) patients in the intervention group and six (50%) in the UC group were registered with the specialist Homeless Health Service Addiction Team, meaning the patients were known to have problem drug or alcohol use and recently received treatment. The proportions of patients registered with specialist homeless vs. mainstream GP practices were comparable between groups. Three patients in the intervention group and two in the UC group were known to the City Ambition Network (CAN) and therefore had a named key worker. CAN is a partnership involving Glasgow city Council, homeless charities and criminal justice, established to identify and support patients who repeatedly or persistently became homeless, or were in frequent contact with criminal justice services.

### Baseline health-care utilisation

Table 3 describes acute services healthcare utilisation, in the 12 month period prior to referral.

**Table 3:** Baseline health care utilisation^a^

	Intervention ***n*** = 12	Usual Care ***n*** = 12
**ED attendance**^**2**^		
**All cause**		
Patients	12 (100)	12 (100)
Attendances/patient year	1.0 (0.6–1.5)	0.8 (0.7–1.6)
**Physical health**		
Patients	11 (92)	10 (83)
Attendances/patient year	0.6 (0.5–0.9)	0.4 (0.1–0.8)
**Mental health**		
Patients	5 (42)	4 (33)
Attendances/patient year	0 (0.0–0.2)	0 (0.0–0.1)
**Addictions**		
Patients	8 (67)	10 (83)
Attendances/patient year	0.2 (0.0–0.5)	0.5 (0.1–0.8)
**Unspecified**		
Patients	0	2 (17)
Attendances/patient year	0.0 (0.0–0.0)	0.0 (0.0–0.0)
**Hospitalisation**		
**All cause**		
Patients	12 (100)	11 (92)
Hospitalisations/patient year	0.6 (0.4–0.8)	0.5 (0.3–0.7)
**Physical health**		
Patients	11 (92)	11 (92)
Hospitalisations/patient year	0 (0.0–0.2)	0 (0.0–0.1)
**Mental health**		
Patients	4 (33)	0
Hospitalisations/patient year	0.0 (0.0–1)	0
**Addictions**		
Patients	8 (67)	8 (67)
Hospitalisations/patient year	0.1 (0.0–0.2)	0.0 (0.0–0.0)
**Scheduled outpatient appointments**		
Patients	8 (67)	8 (67)
Appointments/patient year	4.0 (4.3)	4.3 (6.3)
**Attended**		
Patients	6 (50)	6 (50)
Attendances/patient year	1.0 (1.3)	1.8 (3.4)

^a^ N (%) or median (IQR)

Hospital visits (ED, hospitalisation and out-patient appointments) were comparable between groups in the 12 month period before each patient was referred to PHOENIx. Physical health problems and addictions were the most common reasons for hospitalisations. Most patients (8 (67%) in both groups had scheduled out-patient appointments, with a median of 4 in both groups in the year preceding the intervention; the majority of appointments were missed. Seven (58%) patients in the intervention group and nine (75%) patients in the usual care group had at least one irregular (left hospital against medical advice, without their care plan completed) discharge with a median of 0.1 and 0.2 irregular discharges respectively.

Figure 1 describes patient flow through the service. Of the 12 patients who could not be reached, the team learned (from the SCS Street team) the location of six patients. The remaining six patients could not be traced. All 12 patients who were reached received the intervention as planned. Following the initial engagement, the team made 174 subsequent attempts to engage the patient (speak on phone or meet face to face) (median 12.5 (IQR 5.5–15.8) per patient) in the intervention group leading to 114 consultations (median 7.5 (3.0–14.2) per patient. No patients who were contactable, declined the offer of any PHOENIx team consultations.

The first patient was referred and seen on the same day (19th March 2018); the 24th patient was referred on 6th April 2019, receiving their first health assessment on 24th April 2019. Due to limited availability of the NHS researcher, the censor date was 24th August 2019. Table 4 summarises process outcomes.

**Table 4:** Intervention group: contacts and interventions

	Patients n (%)	Per patient (***n*** = 12) ^a^
Contact attempts	12 (100)	12.5 (5.5–15.8)
Successful contact attempts	12 (100)	7.5 (3–14.3)
Health check including medication review	12 (100)	2.5 (2–3)
Additional clinical assessment		
Physical health	6 (50)	0.5 (0–1)
Mental health	4 (30)	0.0 (0–1)
Addictions	4 (30)	0.0 (0–1)
New diagnosis		
Physical health	6 (50)	0.5 (0–1.8)
Mental health	4 (30)	0.0 (0–1)
Addiction	1 (8.3)	0.0 (0–1)
Prescriptions issued		
New	9 (75)	2.0 (0.3–3.8)
Repeat	11 (91.7)	2.0 (1.3–7.0)
Immediate treatment	7 (58.3)	0.0 (0–1)
Supplies ^b^	9 (75)	3 (0.3–3.8)
Direct referral (appointment +/− transport)		
GP	10 (83.3)	1 (1–2)
Mental health	4 (30)	0 (0–1)
Addictions	6 (50)	0.5 (0–1.8)
Complex needs	7 (58.3)	1 (0–1)
Other e.g. ED	7 (58.3)	1 (0–4.5)
Social visit		
PHOENIx	9 (75)	1.5 (0.3–4.5)
PHOENIx plus other intervention workers	3 (25)	0 (0–0.8)
Appointment reminder	6 (50)	0.5 (0–2)
Benefits / Housing / Advocacy	10 (83.3)	1.5 (1–5)

^a^ Median (IQR) per patient; ^b^Medicines, food, clothes, Gregory Pecks, books etc.

Locations for health checks included the patients’ temporary accommodation or low threshold venues e.g. city centre day centres or evening soup kitchens. The team routinely offered patients clothing, food and drink. Clinics were in the afternoons or evenings. Following clinical assessment, pharmacists made new diagnoses: six patients received new physical health problem diagnoses e.g. deep vein thrombosis. Four had new diagnoses of mental health problems e.g. depression, and one patient had a new diagnoses of opiate addiction. Subsequently, pharmacists prescribed a median of two (IQR 0.3–3.8) new prescriptions per patient. All but one patient received prescriptions for pre-existing repeat medicines. Immediate treatment e.g. for wound care, was received by seven patients. Most patients were offered support for benefits: a median 1.5 interventions (IQR 1–5) per patient were focussed on benefits, housing or advocacy. Complex needs and a high level of multidisciplinary team working is evidenced by the range and number of onward referrals. Because of the difficulties experienced by patients navigating complex health and social care systems, and missing appointments, in most cases, the team organised transport for patients to go to, and return from these appointments, to reduce the burden.

### Health services utilisation outcomes

Outcomes (Table 5) until censor date were collated for each patient using routine NHS data sources. In the intervention group, there were no deaths during follow up (median 5 (range 1–12) months. Two patients in the UC group died of drug/alcohol overdose; reducing the duration of follow up to a median 4 (range 2–7) months.

**Table 5:** Health services utilisation outcomes ^a^

	Intervention ***n*** = 12	Usual care ***n*** = 12
**Emergency Department (ED) (attendances (all cause)**		
Patients	11 (92)	11 (92)
Attendances /patient year	0.7 (0.5–0.9)	1.6 (0.8–4.2)
**Physical health**		
Patients	10 (83)	9 (75)
Attendances/ patient year	0.4 (0.2–0.5)	0.4 (0.1–1.4)
**Mental health**		
Patients	5 (42)	6 (50)
Attendances /patient year	0.0 (0.0–0.2)	0.1 (0.0–0.3)
**Addictions**		
Patients	6 (50)	7 (58)
Attendances /patient year	0.1 (0.0–0.4)	0.3 (0.0–1.0)
**Hospitalisations (all causes)**		
Patients	11 (92)	9 (75)
Hospitalisations /patient year	0.5 (0.4–0.7)	0.6 (0.1–1.3)
**Physical health**		
Patients	9 (75)	7 (58)
Attendances /patient year	0.2 (0.9–0.4)	0.3 (0.0–0.6)
**Mental health**		
Patients	5 (42)	1 (8)
Attendances/patient year	0.1 (0.1–0.1)	0.0 (0.0–0.0)
**Addictions**		
Patients	5 (42)	5 (42)
Attendances/patient year	0.0 (0.0–0.4)	0.0 (0.0–0.3)
**Scheduled outpatient appointments**		
Patients	8 (67)	5 (42)
Appointments/patient year	0.7 (0.18–1.1)	0.0 (0.0–0.5)
**Attended**		
Patients	8 (67)	3 (25)
Attended/patient year	0.2 (0.0–0.8)	0.0 (0.0–0.3)

^a^ N(%) or median (IQR)

Table 5 shows there was a signal of a reduction in the number of ED attendances per patient in the intervention group at follow up: Intervention group 0.7 attendances/patient year (IQR 0.5–0.9) vs. UC 1.6 attendances/patient year (0.8–4.2). The Intervention group ED attendance rate at follow up also represents a reduction compared to baseline (pre-intervention): median number of ED attendances /patient year in the Intervention group at baseline: 1.0 (0.6–1.5) (Table 3) vs 0.7 (0.5–0.9) (Table 5) at follow up. ED attendances/patient year doubled during the study period for patients in the UC group (from 0.8 (0.7–1.6) ED attendances /patient year at baseline (Table 3) to 1.6 (0.8–4.2) attendances/patient year at follow up (Table 5)).

However, there was no difference in the number of patients experiencing ED attendances with 11 patients in each group having at least one ED attendance. This suggests patients in the usual care group had more repeat ED attendances than those in the intervention group. Nine patients from the usual care group were hospitalised compared with 11 in the intervention group; the increase may have been driven by more attendances due to mental or physical health causes, identified by the PHOENIx team during the course of the intervention. Outpatient appointments increased in the intervention group relative to usual care, and patients in the intervention group had increased attendance, which was encouraged and facilitated by the PHOENIx team during and after the intervention.

## Discussion

The aim of the study was to examine the feasibility of recruitment, retention, the extent of intervention delivery and collection of outcome data, prior to a pilot randomised controlled trial. Twenty four people experiencing homelessness were offered, and 24 agreed to participate in the study. Half were subsequently engaged and received serial health checks leading to prescribing, and onward referral. Six of the 12 who were un-contactable, could be traced but their circumstances contra-indicated a visit from the PHOENIx team or researchers suggesting overall recruitment in a subsequent RCT with the need for face to face baseline assessment, would be in the region of 50%, which is similar to one previous study [30]. The sample size of any planned pilot study would therefore require to be doubled. Engagement and retention with the intervention was excellent (all participants in the intervention arm engaged with the team throughout the duration of the study). There was no loss to follow up in terms of accessing participants’ hospital clinical records, providing a low cost approach to collecting follow up acute health service utilisation data in a subsequent pilot study.

Fewer patients had missed appointments in the intervention group with fewer missed appointments per patient, possibly due to the PHOENIx team arranging out patient appointments and supporting travel arrangements. The team also supported patients to attend their GP, although primary health care utilisation data were not sought during this study. The team encouraged patients to go directly to ED where this was deemed necessary, particularly out of hours when GP surgeries were closed. This may account for the slightly higher number of patients with hospitalisations in the intervention group, particularly for mental health problems.

Patients interviewed in a recent qualitative study suggested the PHOENIx team may directly improve health [24]. By design, the current study lacks the power to determine if the intervention does objectively improve health, however our findings (recruitment, retention, uptake of the intervention, extent of data collection) warrant further examination in a randomised controlled pilot study, with parallel health economic and qualitative process evaluation to assess implementation potential.

### Strengths and limitations

Recruitment and attrition difficulties in trials involving people experiencing homelessness is described previously [31–33]. Our recruitment method (AHLT approaching patients in hospital) achieved 100% recruitment of targeted patients, but 50% subsequently unavailable for baseline assessment suggests the need for baseline assessment at the point of recruitment in hospital, or participant incentives e.g. vouchers, or additional approaches e.g. peer recruiters.

Participants’ characteristics were similar to those described in previous studies [13, 15, 18, 34]; increasing the chances of generalisability in different settings and healthcare systems. Findings can be compared to the characteristics of patients experiencing homelessness in Glasgow (Lowrie F et al) [22] and Glasgow/Edinburgh (Zeitler et al) [13]. A comparison of the study sample Table 2 (patient characteristics) with the samples described by Lowrie [22] and Zeitler [13] show similarity in terms of age and sex, but our current sample had a higher prevalence of drug use (92% compared with 62% [22], and 73% [13]), hostel dwelling (60% vs 40% [22]) and rough sleeping (21% vs 13%) [22]. These differences are possibly explained by previous studies sampling exclusively from patients registered with the Homeless Health services in Glasgow and Edinburgh, whereas our inclusion criteria were homelessness and discharged from hospital, 75% of whom had and 25% had no registered GP. Lack of any type of accommodation and not having a registered GP together suggests a lower level of priority attached to, or difficulty associated with GP registration, by patients in our sample.

The complex PHOENIx intervention included components seen as important from the perspective of a person experiencing homelessness e.g. the immediacy of response [24, 35]. It was delivered as planned, with patients receiving a median of seven consultations each following 12 attempts by the PHOENIx team, and no refusals, suggesting acceptability in our sample.

The PHOENIx response to the patient’s health check was tailored, and the patient’s shared primary and secondary care clinical record was updated at each consultation. A wide range of problems were identified and resolved during the consultation. Detailed actions taken as a result of patient consultations, have been described previously [20–22]. Many of the changes to prescribing are evidence based e.g. initiation of an antidepressant for a major depressive episode, and would be expected to reduce the risk of depression causing or contributing to ED attendance. In this feasibility study, by design, limited inferences can be made about the link between prescribing, adherence and health service utilisation outcomes. In a subsequent pilot or definitive study, with a larger sample, if there is a difference in pre-specified outcomes between groups, we would be able to consider how, by prescribing and on occasion, going to a pharmacy and having the medicines dispensed then taken back to patients, adherence would likely improve. In turn, if the prescription was a direct treatment for a specified condition e.g. an infection, or essential to prevent a medical emergency e.g. insulin for a type 1 diabetic, it is likely that subsequent ED visits would be averted in a short time period. The team delivering the intervention were adept at gaining patients’ trust which is known to be important for improving health outcomes [35] and enlisting the support of a wide range of individuals from diverse organisations, to prioritise and co-ordinate care for people with complex needs. The intervention team may have helped to reduce patients’ treatment burden [36]. Treatment burden refers to the demands made of patients by healthcare systems. It includes the work patients have to do to gain an understanding of their health problems, the engagement work they do to access services and treatments (including prescriptions), the work of attending appointments, undergoing investigations and taking medication, as well as self monitoring work they may have to undertake. An example of the treatment burden experienced by people experiencing homelessness, who have a median of 6 or 7 long term conditions, is adhering to complex treatment regimens, or organising already challenging life circumstances to order and re-order their medicines, or attend different, un-co-ordinated services for their mental, physical and addictions long term conditions [36]. Partnership working with GPs is a pre-requisite to co-ordination of primary care: clinical governance for the PHOENIx team rested with the Homelessness Health Service GP service.

Independent Prescriber Pharmacists and Nurses, and SCS street outreach workers are available to provide this intervention beyond the life of the feasibility study, as part of routine service, underscoring opportunities for rollout into routine primary care should the intervention prove beneficial, and cost effective. Outcome measures in this study were relevant, built on previous work, inexpensive to collect, objectively assessed, and reproducible. Surrogate end points could be assessed in subsequent trials, however unlike patients recruited in previous trials of patients recruited because of a single condition, [37–39] people experiencing homelessness have multiple complex health and social care needs meaning outcomes are more diverse. Subsequent pilot work on the PHOENIx intervention could evaluate patient reported outcomes, or effectiveness using health state utilities (for a future cost utility analysis) e.g. through the use of EQ-5D-5L to generate Quality Adjusted Life Years which could then be used alongside cost data to give an indicative picture of cost effectiveness. Two previous UK based studies of patients experiencing homelessness have included economic analyses [40, 41].

It is possible that patients allocated to the usual care group had greater levels of unmet health needs than those who could be reached. Because of the additional difficulty associated with engaging with these patients, they are likely to have had fewer contacts with services e.g. health and social care, addictions, more chaotic lifestyles and perhaps lower prioritisation of health needs and worse health. Randomisation in any subsequent controlled pilot study is needed to reduce selection bias however those who are hard to reach may remain so even within the context of a RCT. In some settings, where the majority of people experiencing homelessness are migrants and low paid workers, and in healthcare systems that are not free at the point of use, our findings may not be generalizable.

Through our pragmatic trial we aim to demonstrate improvements in the quality and reductions in the cost of care. Quality could be measured by patients receiving more appropriate care through being able to access first contact, co-ordinated, continuous care by the PHOENIx team who act as a bridge back into GPs and other specialists in homelessness health [24]. In order to decrease ED visits through an intervention, we need a sufficient number of ED visits for any reduction to be visible. To maximise the chances of an intervention showing a difference between intervention and control groups in a subsequent trial, targeting patients with higher rates of baseline ED presentations may be more appropriate, or those with higher ED presentations due to physical health problems, given the tendency for the team to assess, diagnose and treat physical health problems.

A small number of patients were recruited, but recommendations for sample sizes in feasibility studies are sparse, because the aim is to examine recruitment, retention, intervention fidelity and outcomes [25, 26, 42, 43]. A recent systematic review of RCTs, non RCTs and controlled before-after studies of interventions to improve care of people who are homeless, identified only one feasibility study, with nine participants (six intervention; three control) [19]. More definitive RCTs are needed, including assessment of a wider range of outcomes e.g. quality of life measures, primary care health utilisation, and duration of hospitalisation.

### Comparison with existing literature

In accordance with the recommended stages of complex intervention testing, [25] the PHOENIx intervention was developed and optimised previously [20–22, 24]. The pairing of third sector worker and pharmacist independent prescriber on outreach, as far as we are aware, is a novel approach to improving engagement and uptake of health and social care interventions in people who are homeless [19]. The only other pharmacist led intervention study in this area did not include assessment of wider health needs, prescribing or referral, or offer serial encounters [44].

Systematic and other reviews have described health interventions to improve health in people who are homeless. However there are no known effective and cost effective ‘off the shelf’ interventions involving healthcare professionals [19, 45, 46].

Our target population are people who pay the ultimate price of extreme inequity: they experience severe and multiple disadvantage across their lives and often die sooner because they are worse off. Our findings suggest a pilot study of the intervention would be justified, and thereafter, a definitive randomised controlled trial. If the definitive study shows reduction in emergency health service utilisation, or length of hospitalisation, or fewer deaths in the intervention group with causality attributable to the PHOENIx intervention, then we will have created an intervention to address one aspect of inequity in health for people experiencing homelessness.

The lack of published feasibility and pilot studies preceding definitive RCTs, suggests previous investigators may not have worked through the phases of developing their complex interventions, introducing the possibility of weaknesses in the reporting and conduct of their definitive trials [46, 47]. In terms of generalisability of the study population, observed ED visit rates were lower than those reported in studies from Canada [30, 48] and North America [49]. Multiple factors interact to influence healthcare utilisation in people who are homeless, however the lower rates observed in our study are surprising given the high rates of mental health and substance misuse in our target group [13, 18], which are associated with higher rates of ED use. Differences in access and payment in different health care systems may also explain differential ED rates [50].

Interventions involving tailoring primary care to people who are homeless have been tried previously to decrease ED use [51] or complex interventions which include respite and social care/housing support [52] although not in the context of adequately powered RCTs. The PHOENIx intervention is tailored to individuals over time, combining assessment of health, with housing and opportunities to involve patients in social activities, because housing is an integral component of disease management [53, 54] and having a structure and purpose to daily life is rated as important for patients to remain healthy [53]. The diverse range of outcomes described in this feasibility study suggest the PHOENIx intervention assessed and addressed factors that take precedence over health care in addition to the predisposing needs that drive health seeking behaviour [54]. The social prescribing component of the PHOENIx intervention offered patients an opportunity to move into a place of safety by day and night. We offered patients opportunities to occupy their time with productive, meaningful activities. These activities took patients away from the city centre streets where they ordinarily would spend their time and where drug use and violence was likely. Assessment and intervention to address homelessness led to some patients obtaining accommodation, or better accommodation, giving them a place of relative safety by night, as compared with rough sleeping for example. We suspect most people would regard these components of the intervention as important priorities, in a hierarchy of needs, which underpin good health and may be a pre-requisite to seeking healthcare, at least healthcare that the patient may view as non urgent.

## Conclusions

Trialling a collaborative, pharmacist independent prescriber and third sector homelessness charity worker outreach intervention is feasible in the context of a non randomised trial, suggesting merit in progressing to a randomised controlled pilot study with embedded process and economic evaluation.

## Data Availability

Anonymised data supporting the conclusions of this article are available from the lead author on request.
